# Treatment of Pulpless Teeth

**Published:** 1867-07

**Authors:** J. Smith Dodge

**Affiliations:** the New York College of Dentistry.


### ARTICLE II.

### *Treatment of Pulpless Teeth.*

By Professor J. Smith Dodge, Jr., M. D., D.D.S., of the New York College of Dentistry.

When a man openly advocates heresy, it behooves him to look well to his proofs. The following paper, therefore, will deal much more largely with reasons for the practice it advocates, than with the details of that practice. For, however dentists may differ about the proper treatment of teeth in which the pulp is dead, they pretty thoroughly harmonise in holding that the pulp cavity and canals must be filled with something; and yet the object of this essay is to maintain that they not only *need* not, but *ought* not to be filled at all. The argument must begin with the causes of that which we all aim to avoid, alveolar abcess.

The cases in which alveolar abcess occurs during the life of the pulp, are so few that they may be left, for all practical purposes, out of the account. On the other hand, too, it must probably be expected that under any treatment, as under none, a certain number of cases are doomed to this disease; since some constitutions are so intolerant of irritation, that the mere presence of a pulpless tooth, though in the very best condition, proves soon or late a sufficient exciting cause of inflammation.

Passing by these exceptional cases, I suppose the unanimous sentiment of dentists is that alveolar abcess is caused by the presence *in the pulp-cavity*, of a putrid pulp. In opposition to this view, let us consider a case familiar to us all. Every dentist must have removed old fillings, generally amalgam, from teeth which had never been affected with abcess, and found the pulp cavity filled with a fluid of the most intensely fetid odor. The color of the tooth often shows that the pulp has been long dead, and consequently that this disorganized mass has long filled the cavity. And yet very many cases occur in which no trace of any abcess can be discovered. The presence then, of decayed pulp *in the pulp-cavity*, does not necessarily cause abcess; and so frequent are these cases, in mouths treated during the flourishing days of amalgam, that they afford a very strong presumption, if not a proof, that the disease in question is not caused by dead pulp, so long as this is entirely within the tooth.

It will be objected that such cases are probably fortified by vigor of constitution, as we know some persons are, against periosteal inflammation; but their further history too often refutes this objection. For it is well known that in many such teeth, when the decayed pulp has been removed and the roots filled, alveolar abcess immediately follows; showing that no immunity exists. Why this result is obtained, we shall see as we proceed. It is enough to have shown here that teeth fully predisposed to abcess, often contain a putrid pulp for a long time, without any abcess occurring.

And indeed it would be difficult to tell why poisonous matter in the pulp-cavity should cause inflammation of the periosteum unless it be conceived to pass through the dentine by absorption; in which case it ought to affect the entire membrane, and not merely the part about the apex of the root; especially in upper teeth, whose dependent position must cause the poisonous fluid to gravitate towards the crown.

These considerations lead me to believe that the dead pulp never causes abcess by its presence in the pulp-cavity; but only when by any means a portion of the fetid mass is forced *out of that cavity*, through the foramen at the point of the root, and in this way inoculates the surrounding tissues. If any agencies exist by which this can be done, then we are able to assign a sufficient cause not only for the sequence of alveolar abcess upon death of the pulp, but also for its constant location. Let us examine the agencies by which this might be effected. In the first place, it cannot be doubted that pressure exerted upon the contents of the pulp-cavity, may be transmitted to the structures surrounding the end of the root, in a very appreciable degree. Many teeth have foramina so large that water will run through them in a jet, when injected into the canal of the root. And the same thing may be shown when air alone fills the canal. Every dentist has noticed that he could pass a fine broach into the pulp-canal of certain roots, quite to the apex, without causing the least sensation; while if he wound the same broach with cotton, and then forced it into the same canal—as many do to wipe it out—the patient would complain of acute pain. I have repeatedly alternated the two things in the same tooth, to be quite sure of my position; and the result is uniform. No explanation can be given except that in the latter case the pulp-canal is converted into a syringe, with the broach for its piston; and the air in the root is forced out through the foramen, transmitting to the periosteum the pressure it has received. These facts seem to prove that any force applied to the contents of the pulp-cavity, whether they be liquid or gaseous, may have the effect of extruding a portion of those contents from the root.

Let us consider, in the next place, how this force may be applied to the decomposed pulp. When the pulp of a tooth has been poisoned, and the cavity of decay immediately filled without removing the dead matter within; or when the pulp has died after the external cavity had been closed by filling; an interval of quiet elapses before the symptoms of periosteal inflammation begin. If we open the pulp-cavity, we find that this interval has been sufficient for the disintegration of the pulp, which is removed as a semi-solid or fluid mass; in a word it has putrefied. Now the process of putrefaction is accompanied by a liberation of gases, more or less copious and rapid; and if we conceive the closed pulp-cavity filled with a poisonous fluid mass continually generating gases within itself, we have quite a sufficient agency for the forcing out through the foramen at the apex, of decomposed and poisonous matter. The chemist produces the extreme results of pressure by the evolution of gas within closed vessels.

Nor is this position merely the result of reasoning. Who has not heard his patient express immediate sense of relief, when he cut into the pulp-chamber of a tooth in the first stages of alveolar abcess? And even when the disease has made some progress, so that the tooth is sore and the jaw swollen, great relief is often afforded by drilling a hole either through the filling or through the neck of the tooth, into the pulp-chamber. Facts of this kind show that the process of decomposition in the pulp does produce a sufficient volume of gas to press injuriously through the terminal foramen, upon the tissues surrounding the root. And I believe this, and no other, to be the method by which the decomposing remains of a dead pulp cause alveolar abcess, when they are left undisturbed within the tooth.

But they are not always left undisturbed. Indeed, it sometimes seems as if they might better have been; for, as has been said, abcess often follows promptly after the removal of a fetid pulp, where no sign of disease had previously appeared. And this I think may fairly be laid to the dentist's charge. As the plan of treatment which I am about to propose depends on this conviction, the reader will please give especial attention to this point.

It has been shown that a broach wound with cotton may convert the canal of the root into a tiny syringe, and inject into the periosteum any liquid matter contained in that canal. Suppose now, the root to have been scraped out as thoroughly as is possible, after the pulp has decomposed, and yet a minute drop of fluid to remain in the fine extremity of the canal. The least compression of the air within the tooth must force this drop outward, and inoculate the parts adjacent with a powerful animal poison. I have even seen part of the fine cuttings made by a broach enlarging the canal, forced through the foramen, in a tooth held in the hand to test this point. Of course the compact filling of the pulp-cavity with any material introduced by gradual pressure, must have the same result of ejecting any fluid remaining in the root; and when we remember how often the extremity of the canal is contracted to a capillary size which nothing except a smooth hair-like broach can penetrate, we shall not doubt that the utmost care in cleaning must often leave a small drop of liquid untouched, and I cannot see how the ordinary process can fail to force this out of the foramen. Believing that this occurs frequently, and that it explains many provoking cases of abcess, I have abandoned entirely the filling of roots, and adopted the following rules for my practice.

I. The remains of the pulp must be removed to the utmost possible extent. Mine is no plan for saving labor, and whatever means can effectually remove all decomposed matter, must be used ; provided always they cause no pressure toward the extremity of the roots.

II. When the pulp-cavity is entirely emptied of all organic matter, the cavity of decay, *and that alone,* is to be filled as usual. When it is necessary, to make fast the plug, I carry my filling into the pulp-chamber, but I always prefer to feel, on finishing the operation, that this contains only air.

III. It is absurd to claim that the whole pulp can always be removed. In the roots of molars and sometimes in bicuspids, the position of the decayed cavity or the shape of the canals may compel us to leave considerable portions of dead animal matter. In these cases, I put creosote into the pulp-chamber if I judge it can fairly reach these remains; but I do not force it into the canals. Still, I have but little confidence in the mass becoming so saturated with the antiseptic as to resist putrefaction; and my reliance, in such cases, is on quite another measure. I look forward to the time when this little portion of pulp will begin to ferment and produce gas, and by its pressure force out of the terminal foramen a poisonous drop. The most straightforward means of avoiding this result, seems to be to make a vent by which the accumulating gas may escape more easily than through the pulp-canal of the root. Accordingly, whenever some part of the pulp is unavoidably left within the tooth, I "ventilate" it at the time of filling. It is important to state how this is done. The drill must have a very obtuse V-shape at the point, and be sharpened on both sides. Its cutting end must be a little wider than the shank; and the latter long and flexible. The hole must not be larger than the wire of a medium-sized pin. The dentist must have learned by examination the relative position of the pulp-chamber in various teeth ; and then passing the point of the drill under the free edge of the gum, until it strikes the neck of the tooth beyond the enamel, he can so incline the instrument as to hit the cavity with certainty. It takes some time to perforate the thick wall, and the operator must not be satisfied until he feels the drill plunge suddenly into a vacant space. I do this before filling the tooth; when, if there be any doubt, the perfection of the ventilation can be tested. The gum falls as a curtain over this tiny hole, acting like a valve opening outward.

This operation is not new, but I think no one has explained its necessity, or given the results of long experiment with it until now. I have used it in certain cases for six or seven years, and lately as my regular practice in the cases indicated; and its success is complete. I have never seen the hole, made as directed above, show the least tendency to decay; and I cannot recall a single case in which a tooth treated in this way, and healthy at the time of operating, became affected with abcess afterwards. In one case, some years ago, I extracted a tooth which seemed to have been ventilated and yet had a troublesome abcess, but on breaking it open I found that the drill had not reached the pulp-chamber. Besides, I have known a good many teeth, painful and sore after filling, threatening alveolar abcess, which were relieved by the simple use of the drill, and remained quiet for years. Only a short time ago I saw such a case of seven years' standing, still doing excellent service.

Some readers will laugh at this, and some will rail. But to those who can still learn it is offered as an improved practice, based on sound reasons, and justified by long experience.

---

